# Validation of the EuroSCORE II in a Greek Cardiac Surgical Population: A Prospective Study

**DOI:** 10.2174/1874192401711010094

**Published:** 2017-09-30

**Authors:** G. Stavridis, D. Panaretos, O. Kadda, D. B. Panagiotakos

**Affiliations:** 1 Department of Cardiac Surgery, Onassis Cardiac Surgery Center, Athens, Greece; 2 School of Health Science and Education, Harokopio University, Athens, Greece

**Keywords:** Cardiac surgery, Mortality, Biostatistics, Risk estimation, EuroSCORE

## Abstract

**Objective:**

The objective of this study was to examine the validity of EuroSCORE II in the Greek population.

**Methods:**

A prospective single-center study was performed during November 1, 2013 and November 5, 2016; 621 patients undergoing cardiac surgery were enrolled. The EuroSCORE II values and the actual mortality of the patients were recorded in a special database. Calibration of the model was evaluated with the Hosmer-Lemeshow goodness-of-fit test, and discrimination with the areas under the receiver operating characteristic (ROC) curve.

**Results:**

The observed in-hospital mortality rate was 3% (*i.e.* 18/621 patients). The median EuroSCORE II value was 1.3% (1^st^ quartile: 0.86%, 3^rd^ quartile: 2.46%), which indicates a low in-hospital mortality. Area under the ROC curve for EuroSCORE II was 0.85 (95% CI: 0.75-0.94), suggesting very good correct classification of the patients.

**Conclusion:**

The findings of the present work suggest that EuroSCORE II is a very good predictor of in-hospital mortality after cardiac surgery, in our population and, therefore can safely be used for quality assurance and risk assessment.

## 1 INTRODUCTION

The European System for Cardiac Operative Risk Evaluation (EuroSCORE) was developed between 1995 and 1999 to provide a simple, additive risk model in European adult cardiac surgery population [1, 2]. A total of 19,000 patients from 132 centres and from eight European countries participated in the project. Several validation studies revealed a good predictive ability in different geographical, social and cultural populations. Moreover, the EuroSCORE showed very good performance in various sub-groups of the referent population, as well as for operative techniques that have not been included in the original study [1]. However, EuroSCORE was found to have limitations while some publications demonstrated validation failures and overestimation of the mortality risk [3-5]. Therefore, EuroSCORE became and outdated model for clinical use and patient evaluation. To overcome this problem, an improved tool, the EuroSCORE II, was proposed and became available since October 2011. EuroSCORE II was constructed in the same way as the EuroSCORE, but it was based on data of 22, 381 patients from 154 centers and 43 countries from all around the world prospectively collected over a 12-week period (May-July 2010). The new tool seems to reduce the overestimation of the calculated mortality risk from the EuroSCORE tool [6]. The new in the EuroSCORE II is the definition of mortality used. The old tool predicted the postoperative mortality rate up to 30 days after cardiac surgery, whereas the new model aimed to predict only the in-hospital mortality rate. The main underlying reason for this alteration was the loss of the follow-up data during the first months after operation in the participated centres, which led, according to some opinions, to low-quality data sets [6]. During these years validation studies have shown conflicting results regarding the performance of EuroSCORE II [7]. Moreover, EuroSCORE II has never been validated in Greece, a country with relatively low cardiovascular disease mortality, and with moderate-to-low cardiovascular disease incidence [8]. Thus, the purpose of this study was to evaluate the performance, *i.e.* classification properties, of EuroSCORE II in a Greek cardiac surgery population.

## 2 METHODS

### 2.1 Study Design

A single ‒ center (*i.e.* Onassis Cardiac Center) prospective study was performed; the ethics and scientific Committee of Onassis Cardiac Center approved the design and procedures of the study. Data necessary for calculation of EuroSCORE II were collected prospectively for each patient through their medical records stored in the hospital’s database. The project has not received any funding and the authors declare no conflict of interest.

### 2.2 Study Sample

From November 1, 2013 to November 5, 2016, all 621 consecutive patients (25% female) undergoing major cardiac operations at our hospital were allocated and included in the study. Mean age of the patients was 67 ± 12 years. All patients were operated by the same surgical team.

### 2.3 Measurements

Variables used for the EuroSCORE II calculation were: Age (in years), gender (male/female), renal impairment (normal, moderate, severe, dialysis), pulmonary hypertension, extracardiac arteriopathy, mobility status (poor due to musculoskeletal or neurological dysfunction), previous cardiac surgery, chronic lung disease, active endocarditis, pre-operative state, diabetes mellitus status, New York Heart Association (NYHA) classification, angina at rest, left ventricle function (ejection fraction>50%, 31%-50%, 21%-30%, <20%), recent (within 90 days) myocardial infarction, urgency for the operation (routine admission, urgent, emergency, salvage), weight of the intervention (Coronary Artery Bypass Grafting, valve repair or replacement, replacement of part of the thoracic aorta, repair of a structural defect, maze procedure, resection of a cardiac tumor, or combination). Mortality information was retrieved through hospitals database, and used here as a result variable. Details about the calculation of the EuroSCORE II have been presented freely available to the public at http://www.euroscore.org/calc.html. As proposed by the developers of EuroSCORE II [6], the end-point used in the present analysis was in-hospital all-cause mortality, which was defined as death occurring at any time after surgery during in-hospital period. Additional variables collected were smoking habits (measured as current, former, never, as well as pack-years of smoking), body mass index (measured as weight in Kg divided by height squared, in m^2^), medical record including patient history and management of hypertension, diabetes, dyslipidemia, cardiovascular disease, as well as date of surgery. Moreover, 1-year death rate after hospital discharge was also recorded.

### 2.4 Statistical Analysis

Continuous variables were presented as mean and standard deviation or median and interquartile range when found to follow a skewed distribution. Comparisons of continuous variables between groups were performed using the Student’s t-test (after evaluating equality of variances using the Levene’s test). Categorical variables are presented as frequencies and relative frequencies (percentage) and compared between groups using the chi-square test. Performance of the risk estimation models was assessed via the measurement of calibration and discrimination. Following a logistic regression model, the discriminative power of EuroSCORE II model was estimated by the area under the receiver operating characteristic (ROC) curve, which was calculated as an index to discriminate between survived and died patients after cardiac surgery. The results were presented with 95% confidence interval (CI). The discriminative power of the model was considered good if the area under the curve (AUC) was >0.70. Calibration was evaluated using the Hosmer ‒ Lemeshow goodness-of-fit test and calibration plot of observed and predicted mortality by EuroSCORE II. Statistical calculations were performed using the R package (version 3.3.2, 2016).

## 3 RESULTS

### 3.1 Patients' Characteristics

The pre-operative and intra-operative characteristics of the patients are shown in Table **1**. Most of patients had NYHA functional class I (*i.e.* 71%), good left ventricular (LV) function (67%) and most of them underwent elective heart surgery (98%) for first time (96%).

### 3.2 Observed and Predicted In-hospital Deaths

The observed rate of in-hospital mortality was 18 deaths out of 621 patients (*i.e.* 3%). The median EuroSCORE II value was 1.3 (1^st^ quartile: 0.86, 3^rd^ quartile: 2.46); which means that the in-hospital mortality for the *n* = 621 cardiac surgery patients was estimated to be slightly lower, 1.3%, than the observed and could be classified as low risk (*i.e.* EuroSCORE II values between 1-2). Moreover, based on logistic regression analysis it was revealed that for each one unit increase in the EuroSCORE II the likelihood of in-hospital mortality was increased by 2% (Odds Ratio = 1.02, 95% CI 0.92, 1.13, p <0.001). Overall the correct classification was evident for 605 out of 621 patients, leading to an overall success rate of 97.4%. Based on the logistic regression analysis with EuroSCORE II as an independent factor, the median mortality was 2.0, whereas the observed mortality rate, by quartile of EuroSCORE II, was: 0% of patients in 1^st^ quartile, 1.3% in 2^nd^ quartile, 0.6% of 3rd quartile and 9.6% in 4th quartile (Table **2**).

### 3.3 Accuracy (Discriminative Power)

As shown in Fig. (**1**), the area under the ROC curve (AUC) for the EuroSCORE II was 0.848 (95% CI 0.75 – 0.94, p <0.001), indicating that EuroSCORE II has good discriminative power to distinguish between incidences of patients who died and those who remained alive. Moreover, the accuracy of the EuroSCORE II was 76.8% when an optimal threshold of the score was set to 2.42.

### 3.4 Calibration

The Hosmer-Lemeshow goodness-of-fit test did not show a significant difference between expected and observed mortality according to EuroSCORE II model (Chi-square = 10.9, *p* = 0.21), indicating good calibration of this model in predicting overall in-hospital mortality. Cross-tabulation analysis revealed a slightly underestimation of EuroSCORE II in high-risk deciles and slightly an overestimation in low-risk deciles (Table **3**).

Fig. (**2**) illustrates the age-dependent values of EuroSCORE II in the studied sample. Moreover, in Table **4**, the predicted probabilities of in-hospital death based on EuroSCORE II are presented by smoking (ever) habits, age category and body mass index classification.

### 3.5 Analysis by Patient Group

The aforementioned accuracy and calibration steps were repeated for males and females, smokers and never smokers, overweight/obese and normal weight, aged below or above 60 years, with or without history of cardiovascular disease, and for those with or without previous surgery. The analysis revealed that the AUC for the EuroSCORE II was 0.97 (95% CI 0.95 – 1.00, p = 0.001) in males and 0.62 (95% CI 0.30 - 0.94, p = 0.48) in females (p for difference = 0.08), 0.964 (95% CI 0.92 – 1.00, p = 0.024) in smokers and 0.69 (95% CI 0.43 – 0.94, p = 0.128) in non-smokers (p for difference = 0.08), 0.75 (95% CI 0.54 – 0.97, p = 0.22) in normal weight patients and 0.75 (95% CI 0.43 – 1.00, p = 0.22) in overweight patients (p for difference = 0.99), 0.84 (95% CI 0.68 – 1.00, p = 0.002) in aged above 60 years, 0.84 (95% CI 0.62 – 1.00, p = 0.1) in patients with history of cardiovascular disease and 0.83 (95% CI 0.61 – 1.00, p = 0.02) in patients without history of cardiovascular disease (p for difference = 0.96) and 0.85 (95% CI 0.69 – 1.00, p = 0.003) in patients without previous surgery.

## 4 DISCUSSION

All current guidelines on the management of cardiovascular risk in clinical practice stress the primacy of total risk estimation as the first step in managing individual risk. This is because risk is the product of a number of interacting risk factors. Several risk assessment tools, like EuroSCORE, have been proposed in the past years mainly for primary, as well as for secondary risk prediction, but also for pre- or peri- operative cardiac surgery. However, all these tools have been developed based on certain databases, from specific population or consortia of studies. Thus, their application in other patient groups needs careful evaluation, especially when behavioral, lifestyle or clinical management characteristics are involved in risk prediction. Calibration is a statistical procedure refers to the ability of a risk model to match predicted and observed outcome rates across the entire spread of the data, while discrimination determines how the model distinguishes between groups of people, *e.g.* patients who were alive or who died during an in-hospital period. In the present study, the discriminative power to correctly classify patients as high or low risk and the discriminating validity of EuroSCORE II was evaluated, in Greek patients undergoing major cardiac operations. The data analysis revealed that EuroSCORE II has good calibration and high discriminative power in a Greek surgical population.

The need for pre-operative risk assessment has been underlined in many studies. Since the development of EuroSCORE back in 1990s it has been suggested that it should be considered for calculating risk score for complex cardiac surgical patients. Some other studies similar to the present analyses have been performed in order to evaluate the accuracy of EuroSCORE system into a population. A study by Garcia-Valentin *et al.* [7] included 4034 patients from 20 Spanish centers, evaluated the performance of EuroSCORE II in cardiac surgical patients. The observed mortality rate was 6.5% while predicted mortality rate by EuroSCORE II was 5.7%. ROC curves showed good discriminative ability (AUC = 0.79, 95% CI 0.76 ‒ 0.82) and suggested that EuroSCORE II can be used for quality assurance and risk assessment, as long as a possible slight underprediction of the mortality rate is considered. Di Dedda *et al.* [9], having studied 1090 adult patients, reported that the accuracy of the EuroSCORE II was acceptable, in isolated coronary surgery, and good or excellent for the other operations (AUC: 0.70 ‒ 0.89). The difference between observed (3.75%) and predicted mortality in the overall population was not significant for the EuroSCORE II (3.1%). Similarly, Kieser *et al.* [10], studying 1125 patient undergoing arterial grafting coronary artery bypass graft surgery showed good discrimination for EuroSCORE II while the overall operative mortality was 3.2%. One of the most important findings of the present study was that the mortality rate was 3%, which was similar to that of other studies, in which this value ranged from 1.6% to 6.3% [9-14]. In the study of Borracci *et al.* [11] on the prediction of mortality of 503 patients in Argentina, EuroSCORE II had good discriminative capacity and calibration; in-hospital overall mortality rate was 4.2%, while the mortality rate predicted by the EuroSCORE II was 3.18% (p = 0.402). The latter makes the between populations comparisons feasible and may help scientific community to develop more robust conclusions. Moreover, when the analysis of the present study was stratified by age (below or above 60 years), history of cardiovascular disease and previous cardiac surgery, it demonstrated a good overall discrimination in predicting in-hospital mortality, while discrimination in the gender, smoking status and obesity subgroups, was poorer.

Undoubtedly, the assessment of future events is an evolving and promising area of cardiovascular epidemiology. It has been strongly suggested that operative mortality is a good measure of quality of cardiac surgical care, as long as patient risk factors are taken into consideration. The calculated risk by the EuroSCORE II for a surgical procedure will certainly have clinical consequences for the decision to perform an operation in ‘high-risk’ patients. The clinical implications of these results are important and suggested that the original EuroSCORE is no longer a useful model for clinical risk assessment or quality assurance. EuroSCORE II, as a risk model for mortality calculations was conceived to overcome the performance limitations of its previous versions. During the past years, an increasing number of European hospitals have tested EuroSCORE against other scoring systems, with very good results. Hospital doctors now have an additional tool for initial cardiovascular prevention-especially under the perspective of the current economic crisis, where the cost of treatment must be taken into consideration in decisions about health care provision. The results of this study reveal a useful guide for both quality assurance and surgical risk analysis in daily practice.

## CONCLUSION

The aim of the present study was to test the validity of EuroSCORE II for operative risk during cardiac surgery. Despite the fact that the studied population in this study was from only one center, and therefore cannot be considered as representative for the entire Greek cardiac surgery population, EuroSCORE II was found to be a very good predictor of in-hospital mortality after cardiac surgery, and, therefore can safely be used for quality assurance and risk assessment.

## Figures and Tables

**Fig. (1):**
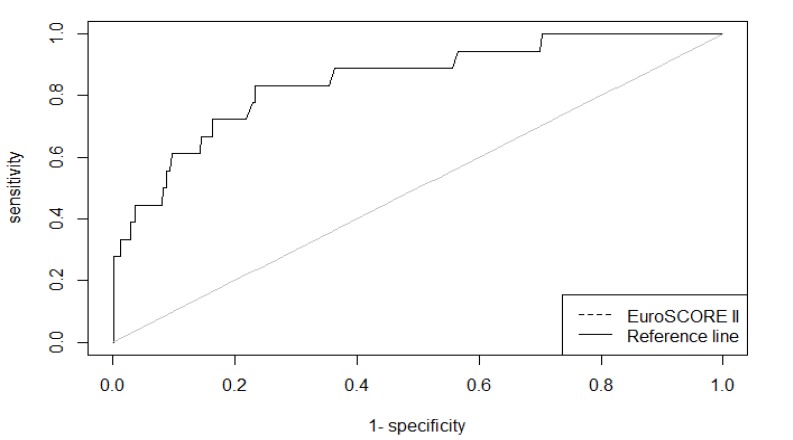
ROC curve for EuroSCORE II of the n = 621 cardiac surgery patients (AUC = 84.8%).

**Fig. (2):**
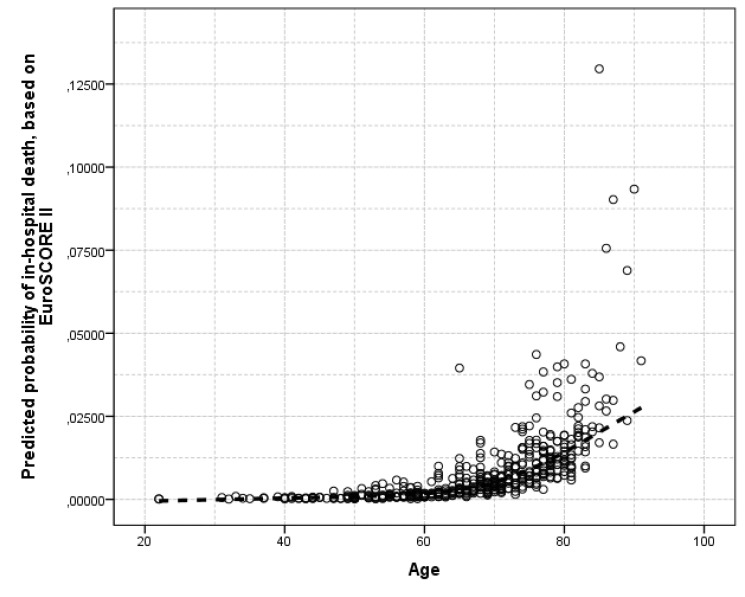
Loess function of EuroSCORE II predicted in-hospital mortality of the n=621 cardiac surgery patients, by age (in years).

**Table 1:** Pre-operative and intra-operative characteristics of the study patients (*n*=621).

**Variables**	
Age (years); mean ± SD (range)	67 ±12 (22 - 91)
Females, n (%)	126 (25%)
Body Mass Index (kg/m^2^); mean ± SD (range)	27.8 ± 4.3 (16.6 ‒ 44.1)
Smoking (Ex-smoker or current smoking); n (%)	275 (59%)
Extra cardiac arteriopathy; n (%)	40 (12.12%)
Poor mobility; n (%)	4 (0.81%)
Diabetes mellitus (insulin-dependent); n (%)	145 (30.14%)
Hypertension; n (%)	352 (73.18%)
Dyslipidemia; n (%)	305 (64.34%)
Family CHD; n (%)	182 (40.44%)
Prior cardiac surgery; n (%)	21 (4.26%)
Chronic Lung Disease; n (%)	41 (8.31%)
Active endocarditis; n (%)	0 (0%)
Critical preoperative condition; n (%)	8 (1.62%)
Angina CCS4, n (%)	3 (2.16%)
Recent myocardial infarction; n (%)	29 (5.9%)
Functional class *NYHA I; n (%)* * NYHA II; n (%)* * NYHA III; n (%)* * NYHA IV; n (%)*	96 (70.59%) 20 (14.7%) 19 (13.97%) 1 (0.73%)
Left ventricular dysfunction; n (%) * EF >50%; n (%)* * EF 31%–50%; n (%)* * EF 21%–30%; n (%)* * EF <21%; n (%)*	226 (67.46%) 97 (28.95%) 9 (2.68%) 3 (0.89%)
Pulmonary hypertension; n (%)	1 (0.2%)
Priority of surgery * Elective; n (%)* * Urgent; n (%)* * Emergency; n (%)* * Salvage; n (%)*	479 (97.75%) 10 (2%) 1 (0.2%) 0 (0%)
Complexity * Isolated CABG; n (%)* * Non CABG; n (%)* * 2 procedures; n (%)* * 3 procedures; n (%)*	179 (36.75%) 180 (36.96%) 100 (20.53%) 28 (5.75%)
Surgery on the thoracic aorta; n (%)	58 (11.76%)
Type of surgery * Isolated CABG; n (%)* * AVR; n (%)* * AAA; n (%)* * Combined; n (%)*	158 (36.32%) 125 (28.73%) 34 (7.82%) 118 (27.13%)
EuroSCORE II; median (range)	1.3 (0.5 – 95)

SD: Standard deviation; n: Number of patients; EuroSCORE: European system for cardiac operative risk evaluation; CCS: Canadian Cardiovascular Society; NYHA: New York Heart Association; EF: Ejection fraction; CHD: Coronary Heart Disease; CABG: Coronary artery bypass grafting; AVR: Aortic valve replacement AAA: Abdominal Aortic Aneurysm

**Table 2:** Observed in-hospital mortality in relation to quartiles estimated by EuroSCORE II.

Outcome	Quartiles of EuroSCORE II			
	[0, 0.86)%	[0.86, 1.3)%	[1.3, 2.44)%	≥2.44%
Alive	152 (100%)	148 (98.7%)	162 (99.4%)	141 (90.4%)
Died	0 (0%)	2 (1.3%)	1 (0.6%)	15 (9.6%)
Total	152	150	163	156

**Table 3:** Classification analysis using Hosmer-Lemeshow goodness-of-fit test for EuroSCORE II of the *n*=621 Greek cardiac surgery patients who participated in the study. Columns present observed and expected cases according to the estimated risk, divided in 10 percentiles groups (1: 0-10%, 2: 11-20%, etc).

Estimated Risk Class	Patients Who Died		Patients Who Were Alive		Total Patients
	Observed	*Expected^1^*	Observed	*Expected^1^*	
0-10%	0	*1.20*	63	*61.80*	63
11-20%	0	*1.22*	63	*61.78*	63
21-30%	1	*1.24*	62	*61.76*	63
31-40%	0	*1.24*	62	*60.76*	62
41-50%	1	*1.26*	61	*60.74*	62
51-60%	0	*1.29*	62	*60.71*	62
61-70%	1	*1.34*	61	*60.66*	62
71-80%	2	*1.40*	59	*59.60*	61
81-90%	4	*1.53*	57	*59.47*	61
91-100%	9	*6.27*	53	*55.73*	62
Overall, *n*	18	*17.99*	603	*603.01*	621

^1^ **Note**: the expected proportion of cases is computed using the coefficients from the applied logistic regression; the Hosmer-Lemeshow test value was 10.9, (*p* = 0.21), suggesting good fit of the observed to the expected values (H_o_: Null hypothesis).

**Table 4:** Estimated, using EuroSCORE II predicted probabilities of in-hospital death, after cardiac surgery (based on a Greek sample of n = 621 patients).

	**Non-smokers**			**Smokers**		
**Body Mass Index**	**< 50 yr**	**50 - 60 yr**	**>60 yr**	**< 50 yr**	**50 - 60 yr**	**>60 yr**
*<25 Kg/m^2^*	0.20%	0.21%	0.26%	0.27%	0.22%	0.22%
*25-30 Kg/m^2^*	0.20%	0.21%	0.22%	0.20%	0.20%	0.34%
*>30 Kg/m^2^*	0.28%	0.21%	0.21%	0.20%	0.21%	0.21%

yr = years old

## References

[1] Roques F., Nashef S.A., Michel P., Gauducheau E., de Vincentiis C., Baudet E., Cortina J., David M., Faichney A., Gabrielle F., Gams E., Harjula A., Jones M.T., Pintor P.P., Salamon R., Thulin L. (1999). Risk factors and outcome in European cardiac surgery: Analysis of the EuroSCORE multinational database of 19030 patients.. Eur. J. Cardiothorac. Surg..

[2] Nashef S.A., Roques F., Michel P., Gauducheau E., Lemeshow S., Salamon R. (1999). European system for cardiac operative risk evaluation (EuroSCORE).. Eur. J. Cardiothorac. Surg..

[3] Kalavrouziotis D., Li D., Buth K.J., Légaré J.F. (2009). The European System for Cardiac Operative Risk Evaluation (EuroSCORE) is not appropriate for withholding surgery in high-risk patients with aortic stenosis: A retrospective cohort study.. J. Cardiothorac. Surg..

[4] Parolari A., Pesce L.L., Trezzi M., Cavallotti L., Kassem S., Loardi C., Pacini D., Tremoli E., Alamanni F. (2010). EuroSCORE performance in valve surgery: A meta-analysis.. Ann. Thorac. Surg..

[5] Smith C.R., Leon M.B., Mack M.J., Miller D.C., Moses J.W., Svensson L.G., Tuzcu E.M., Webb J.G., Fontana G.P., Makkar R.R., Williams M., Dewey T., Kapadia S., Babaliaros V., Thourani V.H., Corso P., Pichard A.D., Bavaria J.E., Herrmann H.C., Akin J.J., Anderson W.N., Wang D., Pocock S.J., Trial Investigators (2011). Transcatheter versus surgical aortic-valve replacement in high-risk patients.. N. Engl. J. Med..

[6] Nashef S.A., Roques F., Sharples L.D., Nilsson J., Smith C., Goldstone A.R., Lockowandt U. (2012). EuroSCORE II.. Eur. J. Cardiothorac. Surg..

[7] Garcia-Valentin A., Mestres C.A., Bernabeu E., Bahamonde J.A., Martín I., Rueda C., Domenech A., Valencia J., Fletcher D., Machado F., Amores J. (2016). Validation and quality measurements for EuroSCORE and EuroSCORE II in the Spanish cardiac surgical population: A prospective, multicentre study.. Eur. J. Cardiothorac. Surg..

[8] Panagiotakos D.B., Georgousopoulou E.N., Fitzgerald A.P., Pitsavos C., Stefanadis C. (2015). Validation of the HellenicSCORE (a Calibration of the ESC SCORE Project) Regarding 10-year risk of fatal cardiovascular disease in Greece.. Hellenic J. Cardiol..

[9] Di Dedda U., Pelissero G., Agnelli B., De Vincentiis C., Castelvecchio S., Ranucci M. (2013). Accuracy, calibration and clinical performance of the new EuroSCORE II risk stratification system.. Eur. J. Cardiothorac. Surg..

[10] Kieser T.M., Rose M.S., Head S.J. (2016). Comparison of logistic EuroSCORE and EuroSCORE II in predicting operative mortality of 1125 total arterial operations.. Eur. J. Cardiothorac. Surg..

[11] Allyn J., Allou N., Augustin P., Philip I., Martinet O., Belghiti M., Provenchere S., Montravers P., Ferdynus C. (2017). A comparison of a machine learning model with EuroSCORE II in predicting mortality after elective cardiac surgery: A decision curve analysis.. PLoS One.

[12] Borracci R.A., Rubio M., Celano L., Ingino C.A., Allende N.G., Ahuad Guerrero R.A. (2014). Prospective validation of EuroSCORE II in patients undergoing cardiac surgery in Argentinean centres.. Interact. Cardiovasc. Thorac. Surg..

[13] Borde D., Gandhe U., Hargave N., Pandey K., Khullar V. (2013). The application of European system for cardiac operative risk evaluation II (EuroSCORE II) and Society of Thoracic Surgeons (STS) risk-score for risk stratification in Indian patients undergoing cardiac surgery.. Ann. Card. Anaesth..

[14] Zhang G.X., Wang C., Wang L., Lu F.L., Li B.L., Han L., Xu Z.Y. (2013). Validation of EuroSCORE II in Chinese patients undergoing heart valve surgery.. Heart Lung Circ..

